# The diagnostic performance of radiomics-based MRI in predicting microvascular invasion in hepatocellular carcinoma: A meta-analysis

**DOI:** 10.3389/fonc.2022.960944

**Published:** 2023-01-31

**Authors:** Gao Liang, Wei Yu, Shuqin Liu, Mingxing Zhang, Mingguo Xie, Min Liu, Wenbin Liu

**Affiliations:** ^1^ Department of Radiology, Hospital of Chengdu University of Traditional Chinese Medicine, Chengdu, Sichuan, China; ^2^ Toxicology Department, West China-Frontier PharmaTech Co., Ltd. (WCFP), Chengdu, Sichuan, China

**Keywords:** hepatocellular carcinoma, microvascular invasion, MRI, radiomics, meta-analysis

## Abstract

**Objective:**

The aim of this study was to assess the diagnostic performance of radiomics-based MRI in predicting microvascular invasion (MVI) in hepatocellular carcinoma (HCC).

**Method:**

The databases of PubMed, Cochrane library, Embase, Web of Science, Ovid MEDLINE, Springer, and Science Direct were searched for original studies from their inception to 20 August 2022. The quality of each study included was assessed according to the Quality Assessment of Diagnostic Accuracy Studies 2 and the radiomics quality score. The pooled sensitivity, specificity, positive likelihood ratio (PLR), negative likelihood ratio (NLR), and diagnostic odds ratio (DOR) were calculated. The summary receiver operating characteristic (SROC) curve was plotted and the area under the curve (AUC) was calculated to evaluate the diagnostic accuracy. Sensitivity analysis and subgroup analysis were performed to explore the source of the heterogeneity. Deeks’ test was used to assess publication bias.

**Results:**

A total of 15 studies involving 981 patients were included. The pooled sensitivity, specificity, PLR, NLR, DOR, and AUC were 0.79 (95%*CI*: 0.72–0.85), 0.81 (95%*CI*: 0.73–0.87), 4.1 (95%*CI*:2.9–5.9), 0.26 (95%*CI*: 0.19–0.35), 16 (95%*CI*: 9–28), and 0.87 (95%*CI*: 0.84–0.89), respectively. The results showed great heterogeneity among the included studies. Sensitivity analysis indicated that the results of this study were statistically reliable. The results of subgroup analysis showed that hepatocyte-specific contrast media (HSCM) had equivalent sensitivity and equivalent specificity compared to the other set. The least absolute shrinkage and selection operator method had high sensitivity and specificity than other methods, respectively. The investigated area of the region of interest had high specificity compared to the volume of interest. The imaging-to-surgery interval of 15 days had higher sensitivity and slightly low specificity than the others. Deeks’ test indicates that there was no publication bias (*P*=0.71).

**Conclusion:**

Radiomics-based MRI has high accuracy in predicting MVI in HCC, and it can be considered as a non-invasive method for assessing MVI in HCC.

## Introduction

Hepatocellular carcinoma (HCC) is the most common primary liver malignant tumor, which is also the third leading cause of cancer death (1, 2). Hepatectomy and liver transplantation are still the main treatments for HCC (3, 4). Despite curative therapies, the prognosis of HCC patients remains poor, with 5-year recurrence rates reaching 50%–70% after hepatectomy and <35% after liver transplantation (5–7). It was proven that 15.0%–57.1% of patients presented microvascular invasion (MVI) after hepatectomy, which is a well-established risk factor for postoperative recurrence (8–10). In addition, the 5-year survival rate of patients with MVI significantly declined (11). For the MVI-positive patients, a wide resection margin is recommended. Therefore, an accurate prediction of MVI before operation is of great importance for clinical treatment decision and prognosis evaluation.

MVI is defined as the cancer cell nest in small vessels lined with endothelium, which is visible only under microscopy (12). Conventional imaging methods are of limited value and pose a challenge for non-invasive diagnosis in assessing MVI in HCC. In recent years, radiomics has been widely applied in the tumor diagnosis, the evaluation of response to treatment, and prognosis prediction. As a new and non-invasive technology, radiomics can high-throughput-extract features from large quantities of images to improve diagnostic or prognostic accuracy, which is also effective to preoperatively predict MVI (13). As imaging markers, the extracted radiomics feature can reflect the microscopic pathological changes of the tumor (**Supplementary Figure S1**), which is promising in the diagnosis of carcinomas (14).

MRI can also provide better soft-tissue resolution, multiparameters, and more stable features for assessing tumor heterogeneity. Previous similar studies have included CT-, MRI-, and US-combined radiomics original studies (13–15). Although they made a subgroup analysis of different imaging modalities, the number of MRI-based radiomics studies included was small. There is no unified conclusion regarding the accuracy of radiomics-based MRI for predicting MVI in HCCs. The current meta-analysis aimed to comprehensively and systematically assess the accuracy of radiomics-based MRI in evaluating the MVI of HCCs.

## Materials and methods

Patients, public-involvement patients, and the public were not involved in this study.

### Searching strategies

The literature search was independently performed by two radiologists. The databases were searched from their inception to 20 August 2022 including PubMed, Cochrane Library, Embase, Web of Science, Ovid MEDLINE, Springer, and ScienceDirect. The search terms were “hepatocellular carcinoma,” “liver malignant tumor,” “liver cancer,” “liver cell carcinoma,” “texture analysis,” “radiomics,” “advanced analysis,” etc. The titles and abstracts were searched for their relevance. Disagreements were discussed and resolved to reach a consensus. In addition, the search strategy is presented in detail in **Supplementary File 1**.

### Study selection

Studies were selected according to the following criteria: (1) original research studies. (2) HCC patients with MVI were confirmed by biopsy or histopathology. (3) Data were available and could be extracted for calculating the true-positive (TP), false-positive (FP), true-negative (TN), and false-negative (FN) values. (4) MRI-based radiomics was applied to predict MVI in HCC. (5) English literature: the excluding criteria were case reports, reviews, abstracts, meta-analyses, insufficient calculable data, or animal studies.

### Data extraction

The relevant information extracted from the original study was as follows: the first author, the year of publication, country and language, sample size, research type, gold standard, the age of patients, TP, FP, FN, TN, MRI field strengths, and radiomics software. When there is a disagreement in the process of document screening and data extraction, the third radiologist will discuss and resolve it.

### Quality assessment of included studies

The quality of each study was assessed on the basis of the Quality Assessment of Diagnostic Accuracy Studies 2 (QUADAS-2) guideline and the radiomics quality score (RQS) (16, 17), which is recommended by the Cochrane collaboration web. The QUADAS-2 tool consists of four parts: (1) patient selection; (2) index test; (3) reference standard; and (4) flow and timing. The RQS checklist is described in **Supplemental Table S1**.

### Statistical analysis

Meta-analysis was performed by Stata version 15.1, and Review Manager software, version 5.3. We adopted a bivariate random effects model to calculate the pooled estimates in advance. The Cochran-Q method and inconsistency index (*I^2^*) were used to investigate heterogeneity among the studies. If *I^2^* > 50%, *P* < 0.05, the observed heterogeneity was significant. If *I^2^* < 50%, *P* > 0.05, the observed heterogeneity was not significant. If there were obvious heterogeneity, the Spearman’s correlation coefficient was used to assess the threshold effect between the sensitivity logit and the specificity logit. If there were no threshold effect, sensitivity analysis and subgroup analysis were performed to further investigate the cause of the heterogeneity.

Pooled sensitivity (Sen), specificity (Spec), PLR, NLR, and DOR were calculated to assess the diagnostic performance of radiomics-based MRI. The summary receiver operating characteristic (SROC) curve was plotted, and the area under the curve (AUC) was calculated. Deeks’ test was used to evaluate publication bias, and P > 0.05, which indicates that there was no significant bias.

### Clinical utility

A Fagan plot was used to evaluate the clinical utility, which demonstrated the posttest probability (P post) of MVI when pretest probabilities were calculated.

## Results

### Research and selection of studies

**Figure 1:**
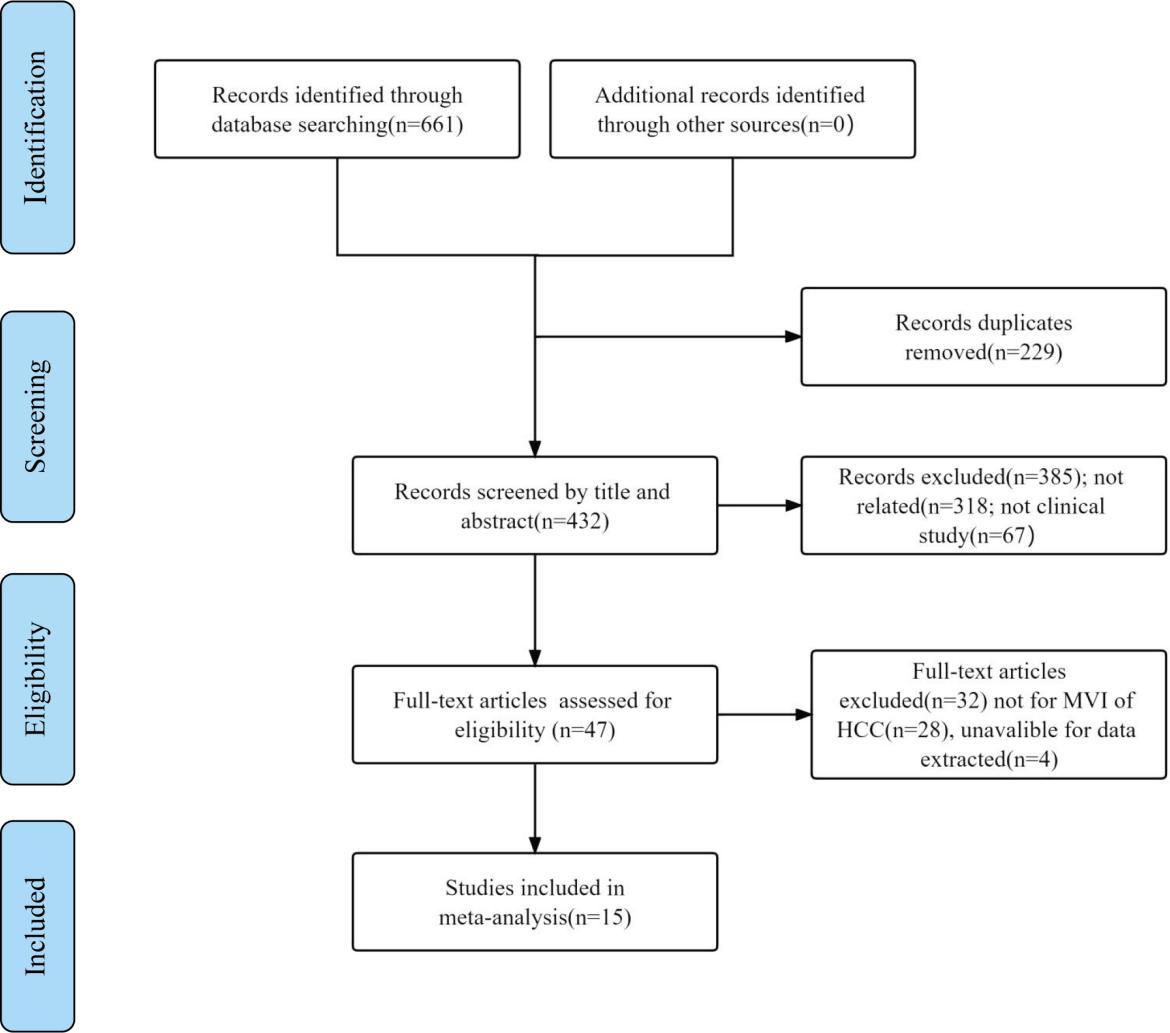
Included study selection process for this meta-analysis.

A total of 661 relevant studies were initially identified from multidatabases, and 229 duplicated articles were excluded. Additionally, 385 records were removed after reading their titles and abstracts and being deemed irrelevant. Subsequently, after reading the full texts, 28 articles were found to be reviews or not related to the MVI of HCC, and 4 articles were unavailable for data extraction. Ultimately, 15 articles were included (18–32). The literature search process is shown in **Figure 1**.

### Study characteristics

The characteristics of the included studies are shown in **Tables 1**, **2**. All 15 studies were retrospective cohort studies. The total number of patients was 981. From the included studies, the number of MVIs and no MVIs were reported and the pathological histology was used as reference standards. Six studies used hepatocyte-specific contrast media (HSCM). The LASSO method and other methods were used as the method for selection in 11 studies and 4 studies, respectively.

**Table 1:** Characteristics of included studies in the meta-analysis.

Author	Year	Country	Study design	Imaging-to-surgery interval	Tumor size (cm), mean (range)	Tumor number	Patient number (all)	(Male/female)	MVI(+)	MVI(-)	MRI parameters		Radiomics software	Gold standard	Data			
											Contrast media	Field strength (T)			TP	FP	FN	TN
Feng (18)	2019	China	Re	Within 1 month	4.3 (2.7, 6.0)	50	50	46/4	20	30	HSCM	3.0	A.K	Histology	18	7	2	23
Zhang R (19)	2019	China	Re	Within 1 month	MVI(+) 5.13 (1.4–10.2), MVI(-) 4.00 (0.8–9.7)	73	73	64/9	26	47	Other	3.0	MATLAB	Histology	21	15	5	32
Chong (20)	2018	China	Re	Within 1 month	within 5.0	106	106	88/18	30	76	Other	1.5	Python	Histology	28	11	2	65
Zhu YJ (21)	2019	China	Re	15 days (range, 7–35 days)	MVI(+)3.82 ±0.88, MVI(-) 3.21 ± 0.94	99	99	32/54	37	62	Other	3.0	Omni-Kinetics	Histology	28	17	9	45
Willson G (22)	2020	USA	Re	Within 3 months	4.5 (2.3–6)	36	36	32/4	22	14	Other	1.5 or 3.0	TexRAD	Histology	15	5	7	9
Zhang Y (23)	2021	China	Re	Within a week	MVI(+)4.00 (2.73–5.00), MVI(-)3.20 (2.00–5.00)	59	59	50/9	34	25	NA	3.0	A.K	Histology	26	5	8	20
Nebbia (24)	2020	USA	Re	Within a week	MVI(+)3.45, MVI(-) 3.84	99	99	83/16	61	38	Other	1.5	Python	Histology	49	8	12	30
Chen Y (25)	2020	China	Re	Within 2 weeks	NA	81	81	NA	33	48	HSCM	3.0	Python	Histology	26	0	7	48
Dai (26)	2020	China	Re	Within a month	MVI(+) 5.54 ± 2.68 (2.3–11.3), MVI(-) 4.49 ± 2.12(1.4–9.2)	69	69	65/4	29	40	Other	3.0	MATLAB	Histology	27	7	2	33
Meng (27)	2021	China	Re	Within a month	3.4 (2.4–4.7)	102	102	84/18	31	71	NA	3.0	Python	Histology	16	9	15	62
Yang Y (28)	2021	China	Re	Within a month	NA	53	53	40/13	26	27	HSCM	1.5 or 3.0	Python	Histology	14	1	12	26
Qu C (29)	2022	China	Re	Within a month	MVI(+) 2.98 ± 1.13, MVI(-)2.94 ± 1.04	53	53	45/8	24	29	Other	3.0	Python	Histology	17	12	7	17
Jiang T (30)	2022	China	Re	NA	MVI(+) 5.70 ± 3.97, MVI(-) 3.91 ± 1.92	21	21	17/4	10	11	HSCM	3.0	R software	Histology	9	1	1	10
Gao L (31)	2022	China	Re	Within a month	NA	35	35	29/6	19	16	HSCM	3.0	R software	Histology	17	4	2	12
Tian Y (32)	2022	China	Re	Within a month	Within 3.0	45	45	35/10	13	32	HSCM	3.0	R software	Histology	11	14	2	18

HSCM, hepatocyte-specific contrast media; NA, not attended; Re, retrospective; A.K, Artificial Intelligent Kit software.

**Table 2:** Radiomic characteristics of included studies in the meta-analysis.

Author	Investigated area	Segmentation method	Feature extraction	Radiomic feature categories	Machine-learning method for feature selection	Number of selected features	AUC of radiomic model with the best performance	AUC of radiomic–clinical model
Feng (18)	VOI: tumor	Manual delineation	1,044 radiomic features	Gray-level histogram, texture analysis, wavelet features	LASSO/LR	10 radiomic features	Training 0.850, Validation 0.833	NA
Zhang R (19)	ROI: tumor and surrounding tissue	Manual delineation	484 radiomic features	Intensity features, texture features, wavelet features	mRMR/LR	mRMR features	Training 0.784, Validation 0.820	Training 0.753, Validation 0.729
Chong (20)	VOI: tumor	Manual delineation	854 radiomic features	Shape, size, intensity, and texture features	LASSO/RF,LR	4 subsets of radiomic features	Training 0.999, Validation 0.918	Training 0.798,Validation 0.725
Zhu YJ (21)	VOI: tumor	Manual delineation	58 texture features	Texture features	LR/texture analysis	10, 12 texture features AP, PP	Training 0.765, Validation 0.773	Training 0.810, Validation 0.794
Willson G (22)	ROI: largest cross section	manual drawn	6 type texture features	Texture features	NA/LR	NA	0.83	NA
Zhang Y (23)	VOI: tumor	Manual segmentation	396 radiomic features	GLCM, GLSZM, RLM, formfactor, haralick features	LASSO/LR	6 subsets of radiomic features	Training 0.889, Validation 0.822	Training 0.901, Validation 0.840
Nebbia (24)	VOI: tumor and margin	Manual segmentation	100 radiomic features	Shape features, first-order features, texture features	LASSO/SVM, decision trees, LR	NA	0.808	NA
Chen Y (25)	VOI: tumor	Manual segmentation	1,395 radiomic features	First-order features, texture features, high-order features	LASSO/SVM, XGBoost, LR	6 subsets of radiomic features	Training 1.00, Validation 0.842	NA
Dai (26)	ROI: axial slice	Manual segmentation	167 radiomic features	Shape features, intensity features, texture features	mRMR,LASSO/RF, SVM, LR	68 radiomic features	0.792	NA
Meng (27)	VOI: tumor	Manually drawn	10,304 radiomic features	Shape features, first-order features, high-order features	LASSO/LR	2,114 radiomic features	0.804	0.872
Yang Y (28)	VOI: tumor and margin	Manual segmentation	851 radiomic features	First-order features, shape features, texture features, wavelet-transformed features	LASSO/mRMR	NA	Training 0.896, Validation 0.788	Training 0.932, Validation 0.917
Qu C (29)	VOI: tumor and margin	Manual segmentation	874 radiomic features	Shape, first-order statistics, GLCM, GLRLM, GLSZM, GLDM	RFE algorithm	560 radiomic feature	Training 0.89, Validation 0.66	Training 0.90, Validation 0.70
Jiang T (30)	ROI: largest cross section	Manual segmentation	1,967 radiomic features	Shapes, first-order statistics, filter-transformed features, GLCM, GLSZM, GLDM, GLCM	LASSO/least absolute shrinkage	11 radiomic features	Training 0.807, Validation 0.835	NA
Gao L (31)	VOI: tumor and margin	Manual segmentation	107 radiomic features	Shape-based characteristics, first-order statistics, textural features	LR, SVC, RFC, adaboost	NA	Training 0.823, Validation 0.740	Training 0.915, Validation 0.868
Tian Y (32)	VOI: tumor and margin	Manual segmentation	1,561 radiomic features	Shape-based features, first-order statistics features, GLCM, GLRLM, GLSZM, GLDM	LASSO/least absolute shrinkage	43 radiomic features	Training 0.842, Validation 0.800	Training 0.934, Validation 0.889

NA, not available; ROI, region of interest; VOI, volume of interest; LASSO, least absolute shrinkage and selection operator; GLCM, gray-level co-occurrence matrix; GLSZM, gray-level size zone matrix; LR, logistic regression; SVM, support vector machine; RLM, run length matrix; mRMR, minimum redundancy maximum relevance; GLRLM, gray-level run length matrix; GLDM, gray-level dependence matrix; RFE, recursive feature elimination; SVC, support vector classifier; RFC, random forest classifier.

### Quality assessment and publication bias

**Figure 2:**
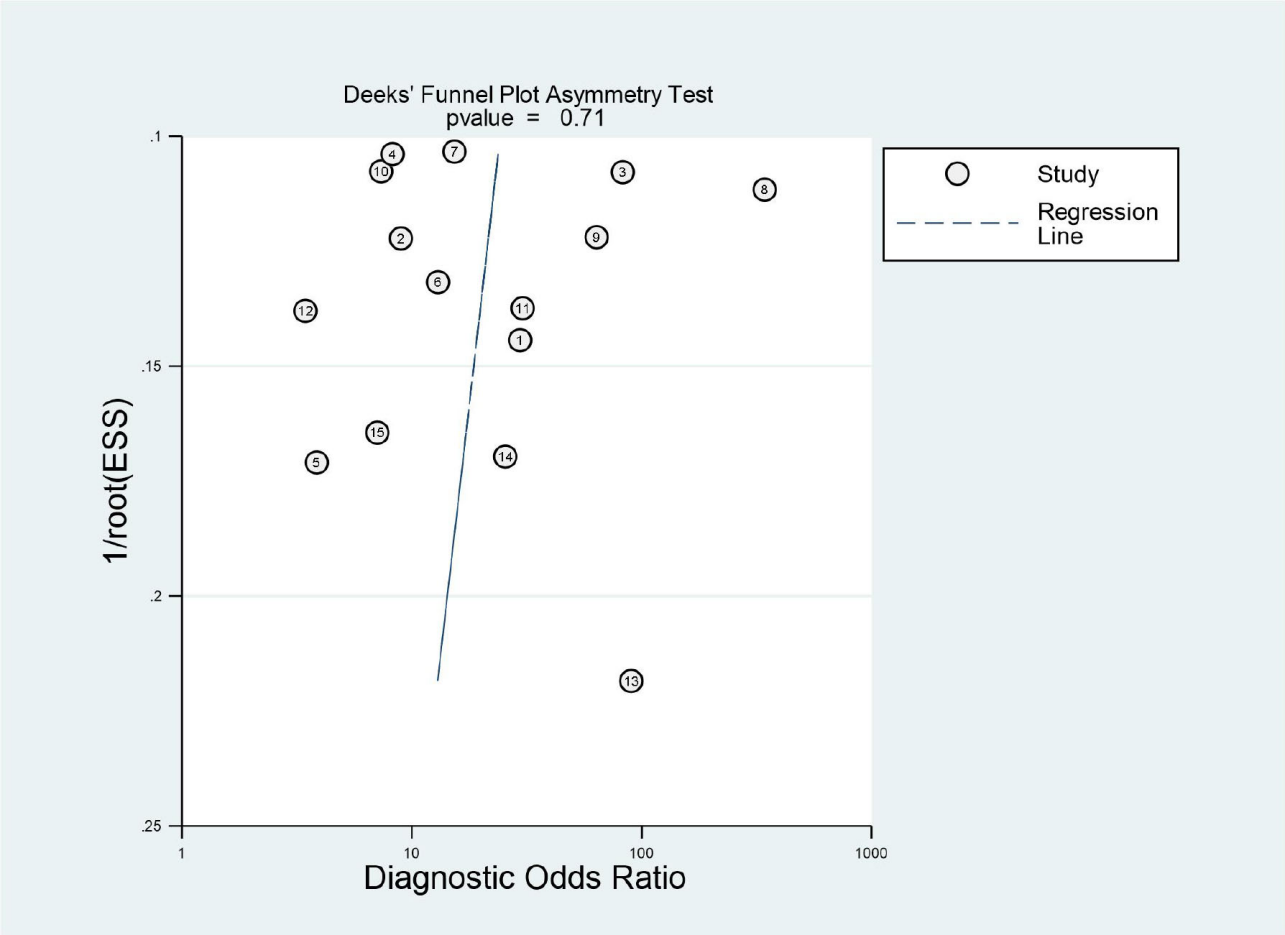
Deeks’ funnel plot to test publication bias.

The quality of the included studies was evaluated according to the QUADAS-2 checklist, and the results are shown in detail in **Table 3**. It was observed that the ‘index test’ in the ‘risk of bias’ and ‘applicability concerns’ revealed uncertain shortcomings, which may suggest bias regarding inclusion. Overall, the quality of all included studies was satisfactory. Deeks’ funnel plot asymmetry test was used to assess the potential publication bias. The results indicated that there was no significant bias (*P* = 0.71), which are shown in **Figure 2**. The 15 studies reached a mean ± standard deviation RQS of 14.80 ± 1.57, median 16, and range 12–17. The average percentage RQS was 20.6% with a maximum of 47.2%. The RQS individual scores and inter-rater agreement are presented in **Supplemental Tables S2, S3**.The RQS was reached with good inter-rater agreement (ICC 0.977, 95% CI 0.934–0.992).

**Table 3:** Results of the Quality Assessment of Diagnostic Accuracy Studies 2 (QUADAS-2) quality assessment of included studies.

Study	Risk of bias			Applicability concerns			
	Patient selection	Index test	Reference standard	Flow and timing	Patient selection	Index test	Reference standard
Feng (18)	+	+	+	+	+	+	+
Zhang.R (19)	+	+	+	+	+	+	+
Chong (20)	+	+	+	+	+	+	+
Zhu YJ (21)	+	+	+	+	+	+	+
Willson G (22)	+	?	+	+	+	+	+
Zhang Y (23)	+	?	+	+	+	+	+
Nebbia (24)	+	+	+	+	+	+	+
Chen Y (25)	+	+	+	+	+	?	+
Dai (26)	+	+	+	+	+	?	+
Meng (27)	+	+	+	+	+	+	+
Yang Y (28)	+	+	+	+	+	+	+
Qu C (29)	+	+	+	+	+	+	+
Jiang T (30)	+	+	+	+	+	?	+
Gao L (31)	+	?	+	+	+	+	+
Tian Y (32)	+	+	+	+	+	+	+

+: Low risk; -: High risk;?: Unclear risk.

QUADAS, Quality Assessment of Diagnostic Accuracy Studies.

### Meta-analysis

**Figure 3:**
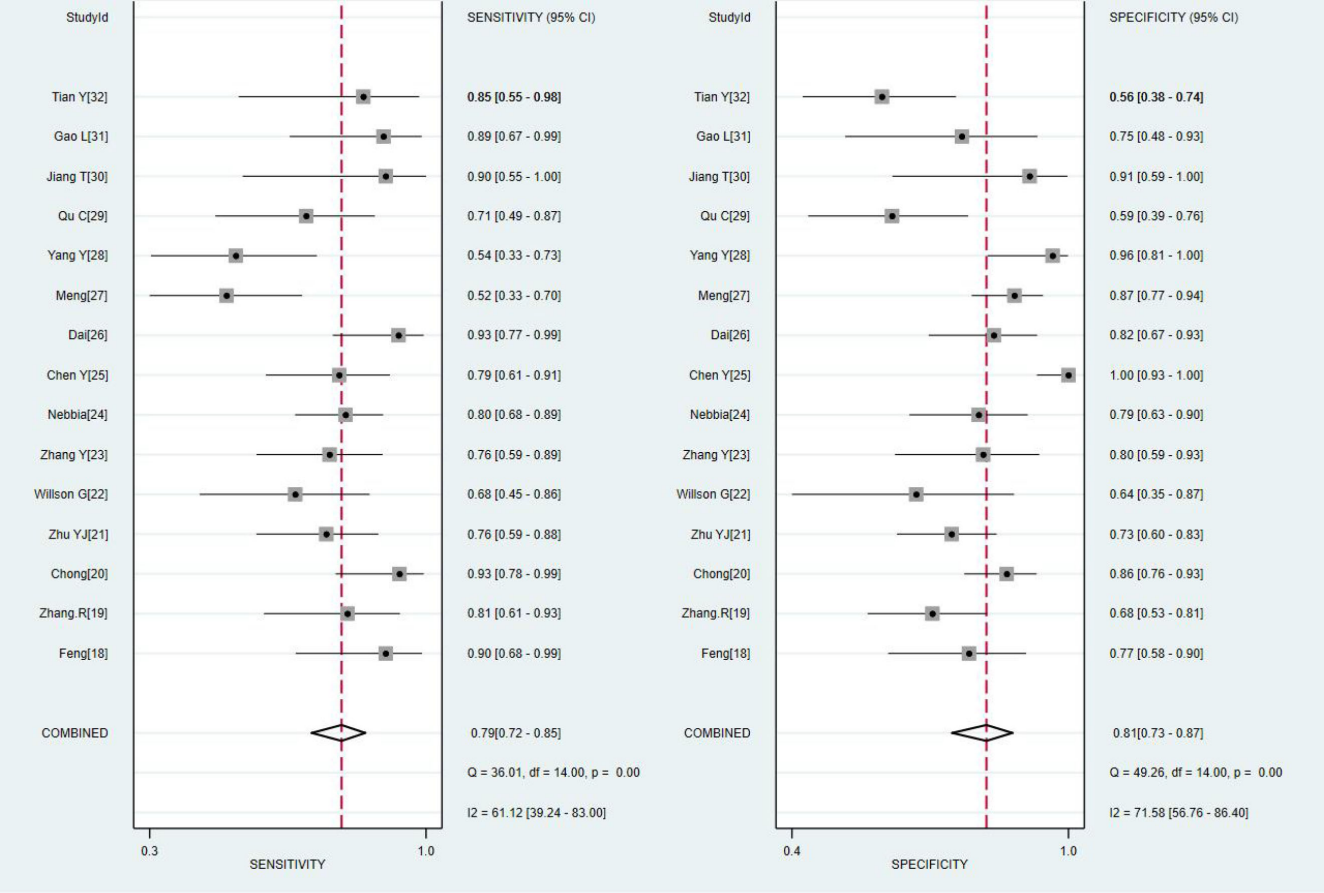
Coupled forest plots of the sensitivity and specificity of radiomics-based microvascular invasion (MRI) for predicting the MVI of hepatocellular carcinoma (HCC).

**Figure 4:**
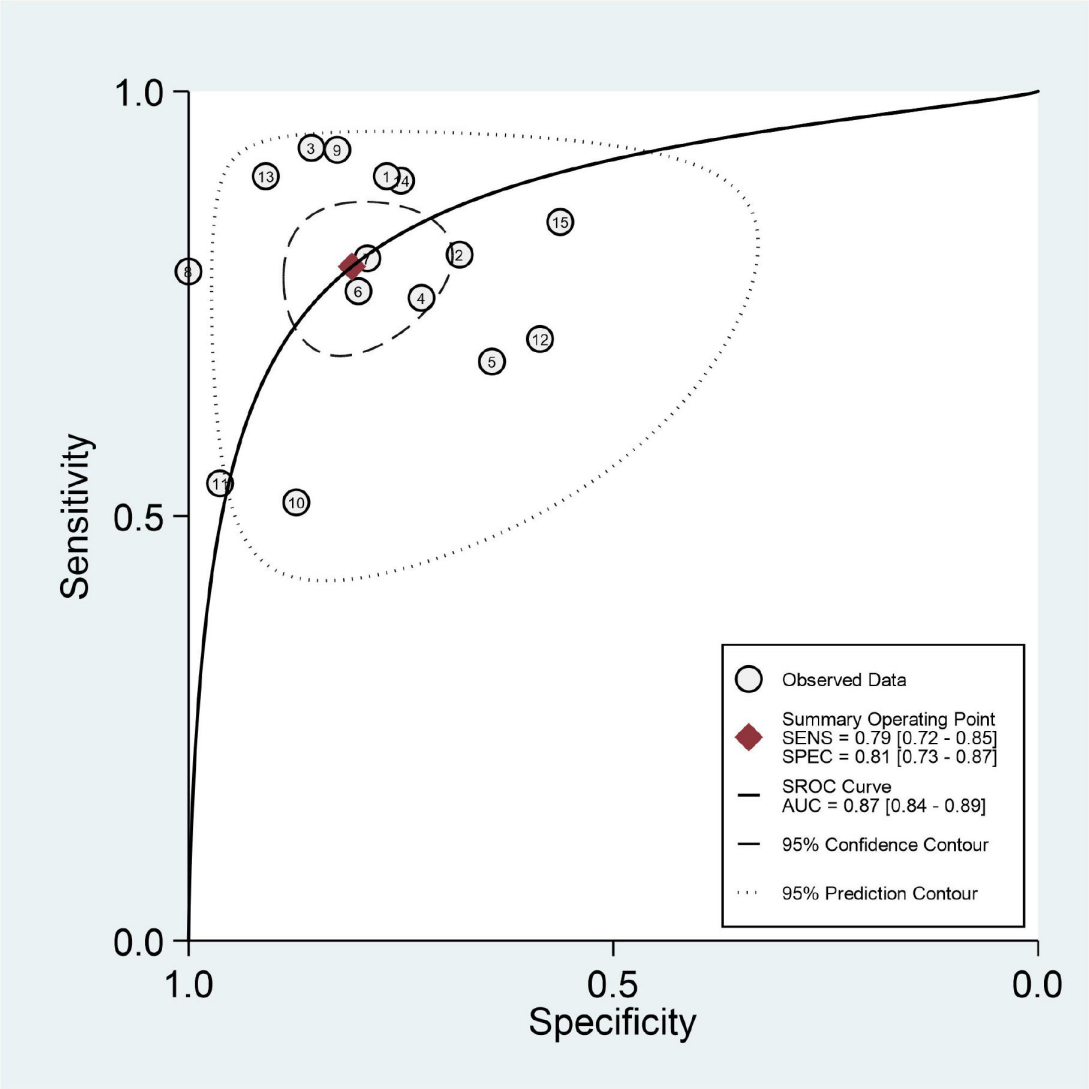
Summary receiver operating characteristic curve to evaluate the MVI of HCC.

The results of the meta-analysis are presented in **Figures 3**, **4**. Pooled sensitivity and specificity were 0.79 (95% *CI* 0.72–0.85) and 0.81 (95% *CI* 0.73–0.87), respectively. The values of PLR, NLR, and DOR were 4.1 (95% *CI* 2.9–5.9), 0.26 (95% *CI* 0.19–0.35), and 16 (95% *CI* 9–28), respectively. The AUC of SROC was 0.87 (95% *CI* 0.84–0.89). These findings indicated that radiomics-based MRI has a high diagnostic performance for evaluating MVI in HCC.

### Exploration of heterogeneity

Heterogeneity was tested using Cochran-Q and *I^2^*. In **Figure 3**, the *P*-value of the Cochran-Q test was 0.00 (*P* < 0.05), and I^2^ was 61.12% in pooled sensitivity. Additionally, the *P*-value of the Cochran-Q test was 0.00 (P < 0.05), and I^2^ was 71.58% in pooled specificity. These results indicated that there was significant heterogeneity in pooled sensitivity and specificity among the included studies.

The result of sensitivity analysis showed that the bivariate model was moderately robust in goodness-of-fit and bivariate normality analyses (**Supplemental Figure S2A, B**). Influence analysis and outlier detection identified two outliers (**Supplemental Figure S2C, D**). After we excluded these outliers, the overall results did not change significantly, which suggested that the results of this study were statistically reliable.

**Figure 5:**
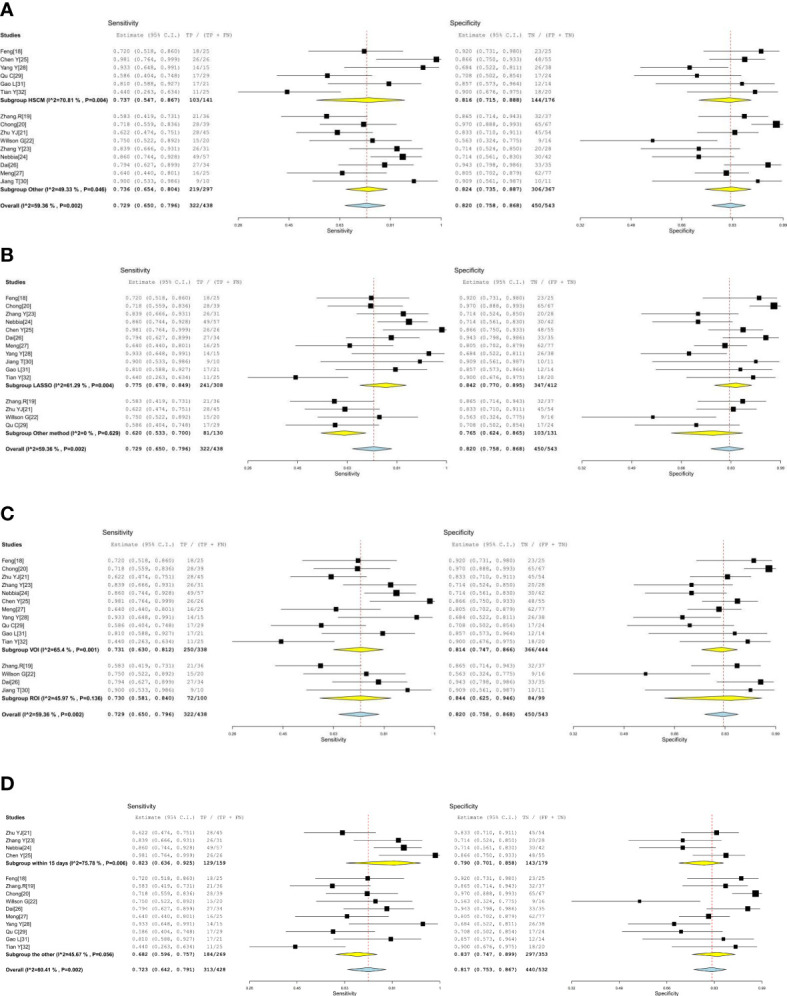
**(A, B)** The forest plots of subgroup analysis. **(C, D)** The forest plots of subgroup analysis.

Subgroup analysis was performed by comparing included studies with different variables. Six studies using HSCM had equivalent sensitivity (0.737 *vs*. 0.729) and specificity (0.816 *vs*. 0.820) compared to nine studies using the other. There were 11 studies with the LASSO method that had high sensitivity (0.775 *vs*. 0.620) and high specificity (0.842 *vs*. 0.765) than other methods. There were 11 studies using the investigated area of VOI that had equivalent sensitivity (0.731 *vs*. 0.730) and low specificity (0.814 *vs*. 0.844) than those studies with ROI. The imaging-to-surgery interval of 15 days had higher sensitivity (0.823 *vs*. 0.682) and slightly low specificity (0.790 *vs*. 0.837) than the others. The details of the subgroup analysis are shown in **Table 4** and **Figures 5A–D**.

**Table 4:** Results of subgroup analysis.

Variate	Studies (n)	Sensitivity (95% CI)	Specificity (95% CI)	PLR	NLR	DOR
Contrast media						
HSCM	6	0.737 (0.547–0.867)	0.816 (0.715–0.888)	4.118 (2.513–6.748)	0.182 (0.059–0.563)	17.769 (5.572–56.079)
Other	9	0.736 (0.654–0.804)	0.824 (0.735–0.887)	3.885 (2.626–5.747)	0.320 (0.239–0.430)	14.027 (8.626–25.021)
Method for the selection of radiomic features						
LASSO	11	0.775 (0.678–0.849)	0.842 (0.770–0.895)	4.719 (3.307–6.734)	0.182 (0.099–0.335)	23.092 (12.505–42.642)
Other methods	4	0.620 (0.533–0.700)	0.765 (0.586–0.865)	2.625 (1.690–4.079)	0.484 (0.344–0.681)	6.042 (3.440–10.611)
Investigated area						
VOI	11	0.731 (0.630–0.812)	0.814 (0.747–0.866)	3.862 (2.681–5.213)	0.228 (0.123–0.421)	14.566 (8.007–26.498)
ROI	4	0.730 (0.581–0.840)	0.844 (0.625–0.946)	4.684 (1.765–12.435)	0.387 (0.246–0.609)	16.222 (4.113–63.984)
Imaging-to-surgery interval						
Within 15 days	4	0.823 (0.636–0.925)	0.790 (0.701–0.858)	3.816 (2.573–5.659)	0.145 (0.045–0.470)	15.291(6.250–37.411)
Other	10	0.682 (0.596–0.757)	0.837 (0.747–0.899)	4.035 (2.631–6.188)	0.327 (0.199–0.538)	13.491(6.699–27.168)

PLR, positive likelihood ratio; NLR, negative likelihood ratio; DOR, diagnostic odds ratio.

### Evaluation of clinical utility

**Figure 6:**
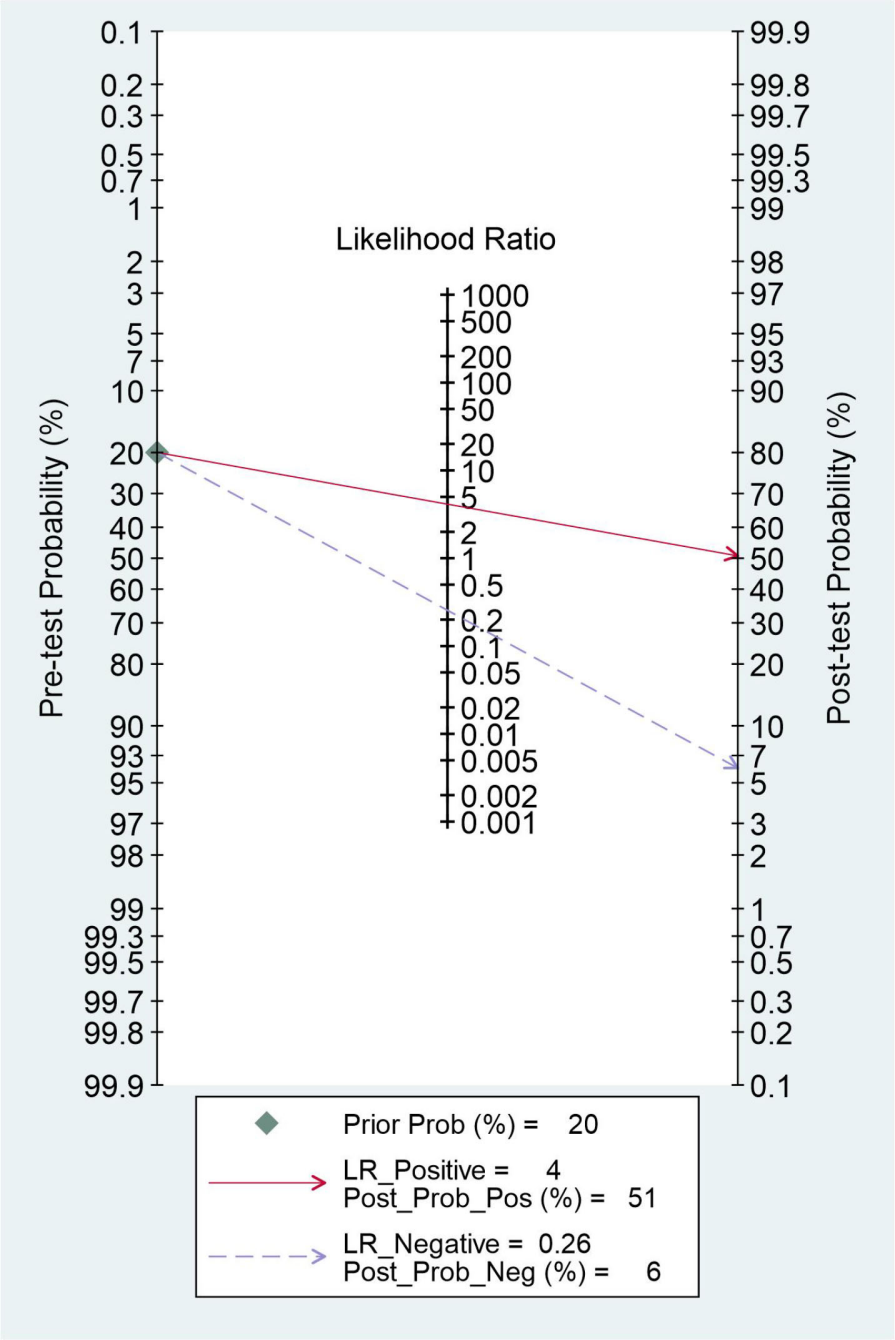
Fagan nomogram for the elucidation of post-test probabilities with a pre-test probability.

The clinical utility of radiomics-based MRI was evaluated by using the likelihood ratio to simulate a Fagan nomogram. The results are shown in **Figure 6**. With a 20% pretest probability of MVI, the posttest probabilities of MVI and given positive and negative results of radiomics-based MRI are 51% and 6%, respectively. The Fagan nomogram revealed that the posttest probability increased by 31% in positive pretest patients but decreased by 14% in patients with a negative pretest, indicating that radiomics based-MRI was useful in clinical practice.

## Discussion

MVI is defined as the presence of cancer cells in the portal vein, hepatic vein, or a large capsular vessel of the surrounding hepatic tissue lined by the endothelium, which is visible on microscopy (12). MVI is recognized as the strongest independent predictor of the early recurrence and poor prognosis of HCC (8–10). Previous studies found that some conventional imaging features, such as the tumor margin, size, number, capsule, shape, apparent diffusion coefficient values, and enhancement pattern, may contribute to the diagnosis of MVI before surgery (33). However, imaging features have some limitations, such as the fact that the reviews of medical images rely on subjective experience. The quantitative radiomics features can reflect the microscopic pathological changes of HCC by extracting features from the overall level of the tumor on the basis of conventional imaging images and evaluating the internal heterogeneity of the tumor (34, 35). Several previous similar studies have demonstrated that radiomics has high accuracy in evaluating the MVI in HCC; however, all of these studies analyzed CT-, MRI-, and ultrasound-based radiomics (13–15). This meta-analysis demonstrates that radiomics-based MRI has high diagnostic performance for predicting the MVI of HCC and can be used as a reliable and quantitative method for the non-invasive diagnosis of MVI in clinical practice. MRI can provide better soft-tissue resolution, multiparameters, and more stable features for assessing tumor heterogeneity.

However, obvious heterogeneity between included studies was noted. HSCM gadoxetate disodium was proven effective to assess the presence of MVI. The study demonstrated that the specificity of the hepatobiliary phase of gadolinium ethoxybenzyl diethylenetriamine pentaacetic acid Gd-EOB-DTPA-enhanced MRI combined with tumor margins and low signal intensity around the tumor to predict MVI is as high as 92.5% (36), but the contrast agent is expensive and not widely used in clinical practice. Subgroup analysis found that different contrast media (HSCM and others), the investigated area, and the method for selection were not the factors of significant heterogeneity. Furthermore, different imaging-to-surgery interval times have different. Therefore, the procedure and method should be standardized by conducting further research.

This study still has some limitations: (1) MRI scanning parameters (including the scanner machine model, field strength, and radiomics software) have not yet been unified; external datasets and different MRI scanning parameters are necessary for confirming the prediction value of the radiomics model. (2) Only English literatures of studies were included, which may result in applicable articles not being included in the review. (3) There was great heterogeneity in pooled estimates between the included studies. All of these factors may reduce the reliability of the results of this study. In the future, a large number of unified and standardized prospective studies are still needed to confirm the value of radiomics based-MRI in predicting the MVI of HCC.

## Conclusion

In conclusion, this study demonstrated that radiomics based on MRI has high accuracy for predicting MVI in HCC, and it can be used as a reliable method to predict the presence of MVI in HCC before surgery in clinical applications.

## Data availability statement

The raw data supporting the conclusions of this article will be made available by the authors, without undue reservation.

## Author contributions

GL and WY have contributed equally to this work and share first Authorship. GL and WY completed manuscript together. GL collected validation group data. SL and MX processed the data and the statistics. ML gave the support of everything we need. All authors did literature researches. All authors contributed to the article and approved the submitted version.
