# Supplementary material for: The diagnostic performance of radiomics-based MRI in predicting microvascular invasion in hepatocellular carcinoma: A meta-analysis
Source: Front Oncol. 2023 Jan 31;12:960944. doi: 10.3389/fonc.2022.960944 (PMC9928182; doi:10.3389/fonc.2022.960944)
Supplement: Supplementary file 1 [file DataSheet_1.pdf]

Search strategy for PubMed (search date: from inception to August 20st, 2022).

#1 Carcinomas, Hepatocellular [Mesh Terms]

#2 (carcinoma*, hepatocellular OR hepatocellular carcinoma* OR liver cancer OR liver cell carcinoma* OR cancer*, liver OR carcinoma*, liver cell OR cell carcinoma, liver OR liver cell carcinoma*)

#3 #1 OR #2

#4 (radiomics OR advanced analysis OR texture analysis) [Title/Abstract]

#5 #3 AND #4

Search strategy for the Cochrane Library (search date: from inception to August 20st, 2022).

#1 [Carcinomas, Hepatocellular] explode all trees

#2 (“carcinoma*, hepatocellular”): ab OR (“hepatocellular carcinoma*”): ab OR (“liver cancer*”): ab OR (“liver cell carcinoma”): ab OR (“cancer*, liver*”): ab OR (“carcinoma*, liver cell”): ab OR (“cell carcinoma, liver”): ab OR (“liver cell carcinoma*”): ab

#3 #1 OR #2

#4 (“texture analysis”): ab OR (“radiomics”): ab OR (“advanced analysis”): ab

#5 #3 AND #4

Search strategy for the EMBASE (search date: from inception to August 20st, 2022). #1 Carcinomas, Hepatocellular /exp

#2 (carcinoma*, hepatocellular OR hepatocellular carcinoma* OR liver cancer OR liver cell carcinoma* OR cancer*, liver OR carcinoma*, liver cell OR cell carcinoma, liver OR liver cell carcinoma*):ti,ab,kw

#3 1-2/OR

#4 (‘texture analysis’ OR ‘radiomics’ OR ‘advanced analysis’): :ti,ab,kw

#5 3-4/AND

Search strategy for Web of Science (search date: from inception to August 20st, 2022) #1 Carcinomas, Hepatocellular[Topic]

#2 (carcinoma*, hepatocellular OR hepatocellular carcinoma* OR liver cancer OR liver cell carcinoma* OR cancer*, liver OR carcinoma*, liver cell OR cell carcinoma, liver OR liver cell carcinoma*) [Title/Abstract]

#3 #1 OR #2

#4 (texture analysis OR radiomics OR advanced analysis) [Title/Abstract]

#5 #3 AND #4

Search strategy for the OVID Medline (search date: from inception to August 20st, 2022).

#1 exp Carcinomas, Hepatocellular.mp.

#2 exp carcinoma*, hepatocellular/ or exp hepatocellular carcinoma/ or exp liver cancer/ or exp liver cell carcinoma*/ or exp cancer*, liver/ or exp carcinoma*, liver cell/ or exp cell carcinoma, liver/ or exp liver cell carcinoma*.mp

#3 #1 OR #2

#4 texture analysis.mp. or exp radiomics/ or exp advanced analysis.mp.

#5 #3 AND #4

Search strategy for the ScienceDirect (search date: from inception to August 20st, 2022).

#1 (carcinoma*, hepatocellular OR hepatocellular carcinoma* OR liver cancer OR liver cell carcinoma* OR cancer*, liver OR carcinoma*, liver cell OR cell carcinoma, liver OR liver cell carcinoma*) [Full text]

#2(“texture analysis” OR “radiomics” OR "advanced analysis") [Full text]

#3 #1 AND #2

Search strategy for the Springer (search date: from inception to August 20st, 2022).(((“carcinoma*, hepatocellular” OR “hepatocellular carcinoma*” OR “liver cancer” OR “liver cell carcinoma*” OR “cancer*, liver” OR “carcinoma*, liver cell” OR “cell carcinoma, liver” OR “liver cell carcinoma*” AND (“texture analysis” OR “advanced analysis”)))
