# Supplementary figures and images for: The diagnostic performance of radiomics-based MRI in predicting microvascular invasion in hepatocellular carcinoma: A meta-analysis
Source: Front Oncol. 2023 Jan 31;12:960944. doi: 10.3389/fonc.2022.960944 (PMC9928182; doi:10.3389/fonc.2022.960944)

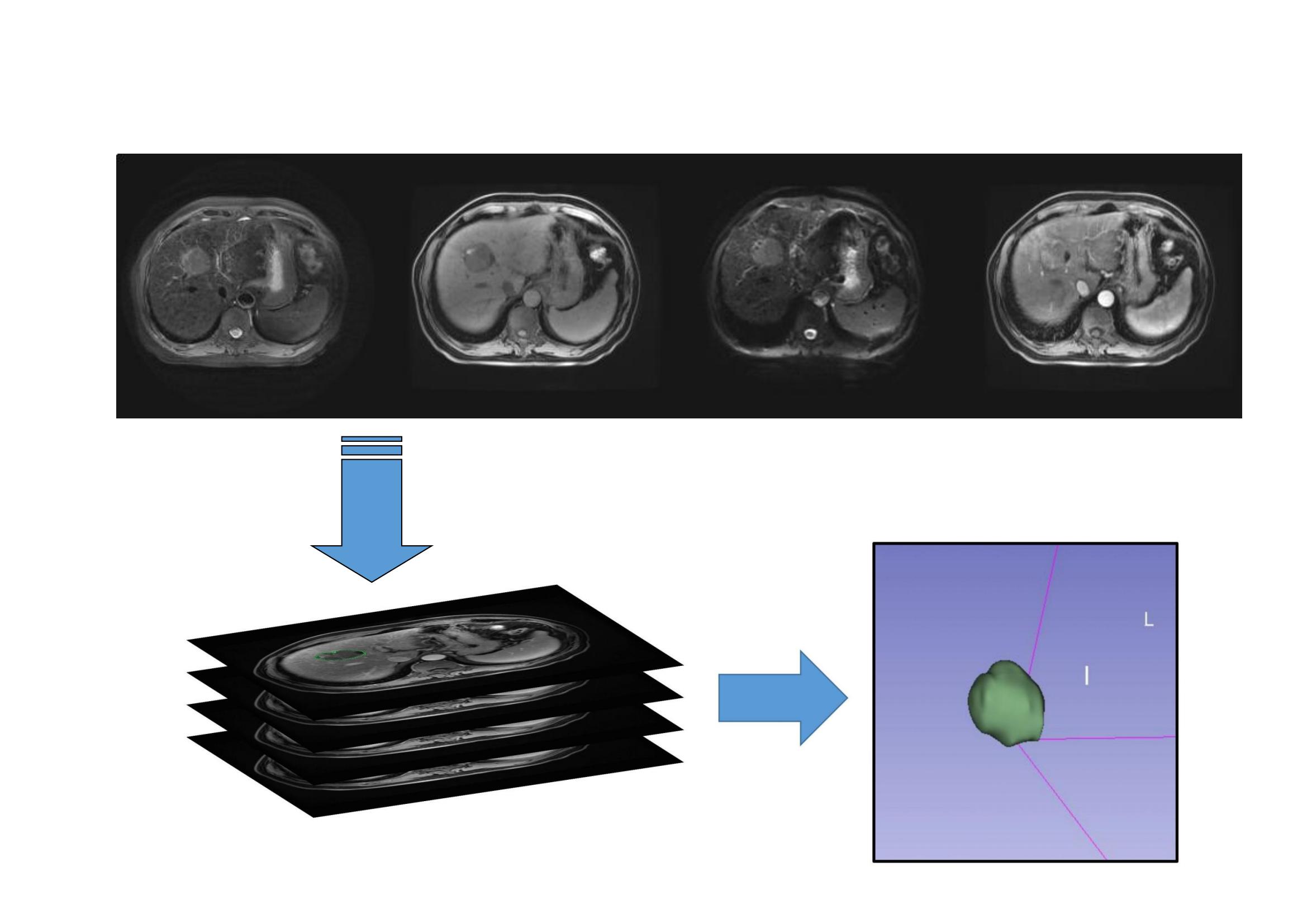

Supplement: Supplementary file 2 [file Image_1.jpeg]

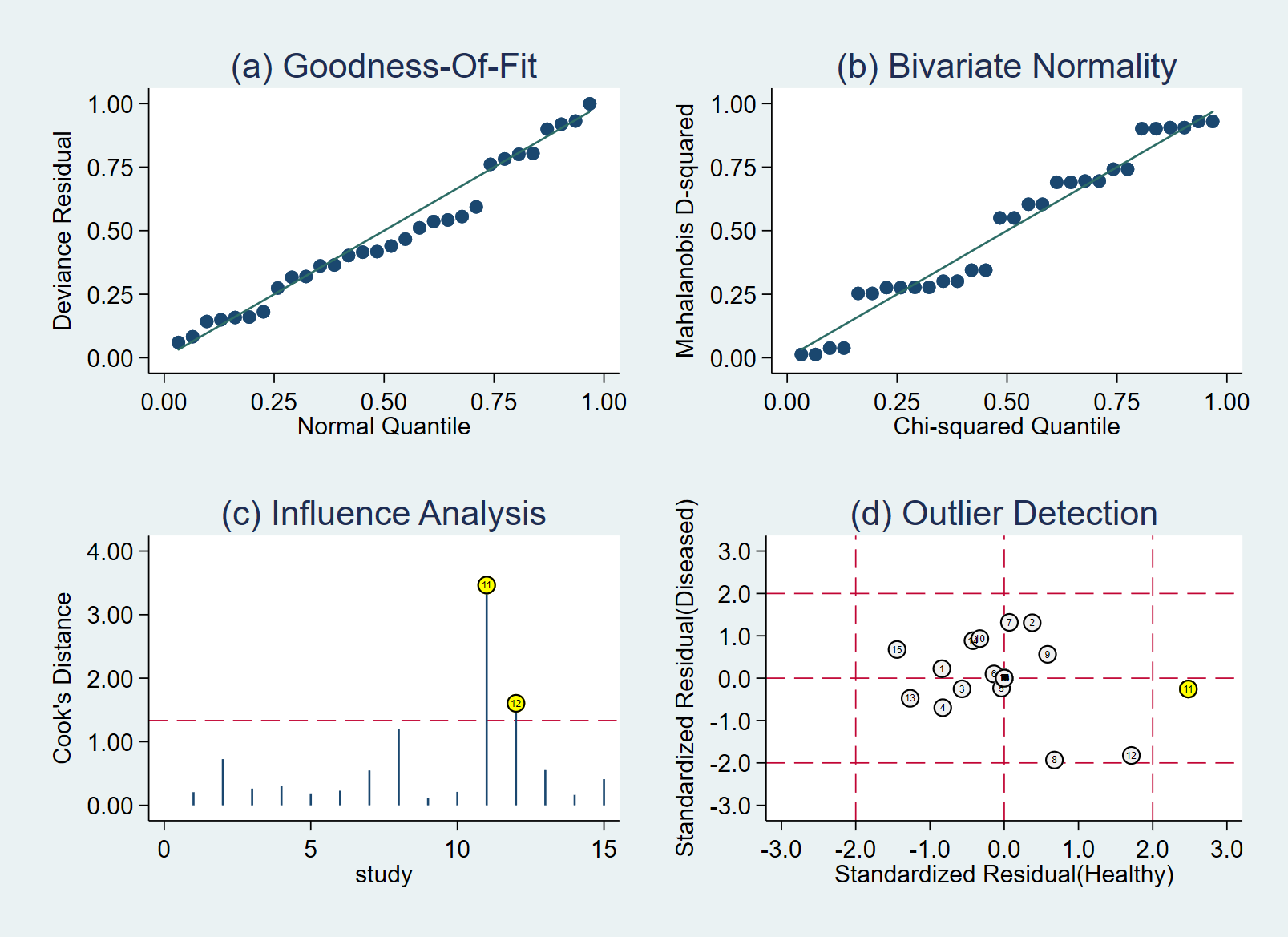

Supplement: Supplementary file 3 [file Image_2.tif]
