# Supplementary material for: The diagnostic performance of radiomics-based MRI in predicting microvascular invasion in hepatocellular carcinoma: A meta-analysis
Source: Front Oncol. 2023 Jan 31;12:960944. doi: 10.3389/fonc.2022.960944 (PMC9928182; doi:10.3389/fonc.2022.960944)
Supplement: Supplementary file 4 [file Table_1.doc]

| Table S1 Elements of the RQS | | |
| --- | --- | --- |
| **No.** | **RQS scoring item** | **Points and Interpretation** |
| 1 | **Image protocol quality** - well-documented image protocols (for example, contrast, slice thickness, energy, etc.) and/or usage of public image protocols allow reproducibility/replicability | + 1 if protocols are well-documented  + 1 if public protocol is used |
| 2 | **Multiple segmentations** - possible actions are: segmentation by different physicians/algorithms/software, perturbing segmentations by (random) noise, segmentation at different breathing cycles. Analyse feature robustness to segmentation variabilities | + 1 if segmented multiple times (different physicians, algorithms, or perturbation of regions of interest) |
| 3 | **Phantom study on all scanners** - detect inter-scanner differences and vendor-dependent features. Analyse feature robustness to these sources of variability | + 1 if texture phantoms were used for feature robustness assessment |
| 4 | **Imaging at multiple time points** - collect images of individuals at additional time points. Analyse feature robustness to temporal variabilities (for example, organ movement, organ expansion/ shrinkage) | + 1 multiple time points for feature robustness assessment |
| 5 | **Feature reduction or adjustment for multiple testing** - decreases the risk of overfitting. Overfitting is inevitable if the number of features exceeds the number of samples. Consider feature robustness when selecting features | - 3 if neither measure is implemented  + 3 if either measure is implemented |
| 6 | **Multivariable analysis with non-radiomics features** (for example, EGFR mutation) - is expected to provide a more holistic model. Permits correlating/inferencing between radiomics and non-radiomics features | + 1 if multivariable analysis with non-radiomics features |
| 7 | **Detect and discuss biological correlates** - demonstration of phenotypic differences (possibly associated with underlying gene–protein expression patterns) deepens understanding of radiomics and biology | + 1 if present |
| 8 | **Cut-off analyses** - determine risk groups by either the median, a previously published cut-off or report a continuous risk variable. Reduces the risk of reporting overly optimistic results | + 1 if cutoff either pre-defined or at median or continuous risk variable reported |
| 9 | **Discrimination statistics** - report discrimination statistics (for example, C-statistic, ROC curve, AUC) and their statistical significance (for example, p-values, confidence intervals). One can also apply resampling method (for example, bootstrapping, cross-validation) | + 1 if a discrimination statistic and its statistical significance are reported  + 1 if a resampling method technique is also applied |
| 10 | **Calibration statistics** - report calibration statistics (for example, Calibration-in-the-large/slope, calibration plots) and their statistical significance (for example, *P*-values, confidence intervals). One can also apply resampling method (for example, bootstrapping, cross-validation) | + 1 if a calibration statistic and its statistical significance are reported  + 1 if a resampling method technique is also applied |
| 11 | **Prospective study registered in a trial database** - provides the highest level of evidence supporting the clinical validity and usefulness of the radiomics biomarker | + 7 for prospective validation of a radiomics signature in an appropriate trial |
| 12 | **Validation** - the validation is performed without retraining and without adaptation of the cut-off value, provides crucial information with regard to credible clinical performance | − 5 if validation is missing  + 2 if validation is based on a dataset from the same institute/  + 3 if validation is based on a dataset from another institute/  + 4 if validation is based on two datasets from two distinct institutes/  +4 if the study validates a previously published signature/  +5 if validation is based on three or more datasets from distinct institutes  *Datasets should be of comparable size and should have at least 10 events per model feature |
| 13 | **Comparison to ‘gold standard**’ - assess the extent to which the model agrees with/is superior to the current ‘gold standard’ method (for example, TNM-staging for survival prediction). This comparison shows the added value of radiomics | + 2 for comparison to gold standard |
| 14 | **Potential clinical utility** - report on the current and potential application of the model in a clinical setting (for example, decision curve analysis) | + 2 for reporting potential clinical utility |
| 15 | **Cost-effectiveness analysis** - report on the cost-effectiveness of the clinical application (for example, QALYs generated) | + 1 for cost-effectiveness analysis |
| 16 | **Open science and data** - make code and data publicly available. Open science facilitates knowledge transfer and reproducibility of the study | + 1 if scans are open source  + 1 if region of interest segmentations are open source  + 1 if code is open source  + 1 if radiomics features are calculated on a set of representative ROIs and the calculated features and representative ROIs are open source |
| Total points (36 = 100%) | | |

| Table S2. Individual scores of RQS (GL /WY) | | | | | | | | | | | | | | | | | | | | | | | | | | | | | | | | |
| --- | --- | --- | --- | --- | --- | --- | --- | --- | --- | --- | --- | --- | --- | --- | --- | --- | --- | --- | --- | --- | --- | --- | --- | --- | --- | --- | --- | --- | --- | --- | --- | --- |
| **Author** | **Image Protocol** | | **Multiple Segmentations** | | **Phantom Study** | | **Multiple Time Points** | | **Feature Reduction** | | **Non Radiomics** | | **Biological Correlates** | | **Cut-off** | | **Discrimination and Resampling** | | **Calibration** | | **Prospective** | | **Validation** | | **Gold Standard** | | **Clinical Utility** | | **Cost-effectiveness** | | **Open Science** | |
| **Feng[18]** | 1 | 1 | 1 | 1 | 0 | 0 | 0 | 0 | 3 | 3 | 0 | 0 | 0 | 0 | 0 | 0 | 2 | 2 | 0 | 0 | 0 | 0 | 2 | 2 | 2 | 2 | 2 | 2 | 0 | 0 | 1 | 1 |
| **Zhang.R[19]** | 1 | 1 | 1 | 1 | 0 | 0 | 0 | 0 | 3 | 3 | 1 | 1 | 0 | 0 | 0 | 0 | 2 | 2 | 0 | 0 | 0 | 0 | 2 | 2 | 2 | 2 | 2 | 2 | 0 | 0 | 1 | 1 |
| **Chong[20]** | 1 | 1 | 1 | 1 | 0 | 0 | 0 | 0 | 3 | 3 | 0 | 0 | 0 | 0 | 1 | 1 | 2 | 2 | 0 | 0 | 0 | 0 | 2 | 2 | 2 | 2 | 2 | 2 | 0 | 0 | 1 | 1 |
| **Zhu YJ[21]** | 1 | 1 | 1 | 1 | 0 | 0 | 0 | 0 | 3 | 3 | 1 | 1 | 1 | 0 | 1 | 1 | 2 | 2 | 0 | 0 | 0 | 0 | 2 | 2 | 2 | 2 | 0 | 0 | 0 | 0 | 1 | 1 |
| **Willson G[22]** | 1 | 1 | 1 | 1 | 0 | 0 | 0 | 0 | 3 | 3 | 1 | 1 | 0 | 0 | 1 | 1 | 2 | 2 | 0 | 0 | 0 | 0 | 2 | 2 | 2 | 2 | 0 | 0 | 0 | 0 | 1 | 1 |
| **Zhang Y[23]** | 1 | 1 | 1 | 1 | 0 | 0 | 0 | 0 | 3 | 3 | 1 | 1 | 0 | 0 | 1 | 1 | 2 | 2 | 0 | 0 | 0 | 0 | 2 | 2 | 2 | 2 | 2 | 2 | 0 | 0 | 1 | 1 |
| **Nebbia[24]** | 1 | 1 | 1 | 1 | 0 | 0 | 0 | 0 | 3 | 3 | 0 | 0 | 0 | 0 | 0 | 0 | 2 | 2 | 0 | 0 | 0 | 0 | 2 | 2 | 2 | 2 | 0 | 0 | 0 | 0 | 1 | 1 |
| **Chen Y[25]** | 1 | 1 | 1 | 1 | 0 | 0 | 0 | 0 | 3 | 3 | 0 | 0 | 0 | 0 | 1 | 1 | 2 | 2 | 0 | 0 | 0 | 0 | 2 | 2 | 2 | 2 | 0 | 0 | 0 | 0 | 1 | 1 |
| **Dai[26]** | 1 | 1 | 1 | 0 | 0 | 0 | 0 | 0 | 3 | 3 | 0 | 0 | 0 | 0 | 0 | 0 | 2 | 2 | 0 | 0 | 0 | 0 | 2 | 2 | 2 | 2 | 0 | 0 | 1 | 1 | 1 | 1 |
| **Meng[27]** | 1 | 1 | 1 | 1 | 0 | 0 | 0 | 0 | 3 | 3 | 0 | 0 | 0 | 0 | 0 | 0 | 2 | 2 | 0 | 0 | 0 | 0 | 2 | 2 | 2 | 2 | 2 | 2 | 0 | 0 | 1 | 1 |
| **Yang Y[28]** | 1 | 1 | 1 | 1 | 0 | 0 | 0 | 0 | 3 | 3 | 1 | 1 | 0 | 0 | 1 | 1 | 2 | 2 | 0 | 0 | 0 | 0 | 2 | 2 | 2 | 2 | 2 | 2 | 0 | 0 | 1 | 1 |
| **Qu C[29]** | 1 | 1 | 1 | 1 | 0 | 0 | 0 | 0 | 3 | 3 | 1 | 1 | 0 | 0 | 1 | 1 | 2 | 2 | 0 | 0 | 0 | 0 | 2 | 2 | 2 | 2 | 2 | 2 | 0 | 0 | 1 | 1 |
| **Jiang T[30]** | 1 | 1 | 1 | 1 | 0 | 0 | 0 | 0 | 3 | 3 | 1 | 1 | 0 | 0 | 1 | 1 | 2 | 2 | 0 | 0 | 0 | 0 | 2 | 2 | 2 | 2 | 2 | 2 | 0 | 0 | 1 | 1 |
| **Gao L[31]** | 1 | 1 | 1 | 1 | 0 | 0 | 0 | 0 | 3 | 3 | 1 | 1 | 0 | 0 | 1 | 1 | 2 | 2 | 0 | 0 | 0 | 0 | 2 | 2 | 2 | 2 | 2 | 2 | 0 | 0 | 1 | 1 |
| **Tian Y[32]** | 1 | 1 | 1 | 1 | 0 | 0 | 0 | 0 | 3 | 3 | 1 | 1 | 0 | 0 | 0 | 0 | 2 | 2 | 0 | 0 | 0 | 0 | 4 | 4 | 2 | 2 | 2 | 2 | 0 | 0 | 1 | 1 |
| **Defined range** | 0 – 2 | | 0 – 1 | | 0 – 1 | | 0 – 1 | | -3 – 3 | | 0 – 1 | | 0 – 1 | | 0–1 | | 0 – 2 | | 0 – 1 | | 0 – 7 | | -5 – 5 | | 0 – 2 | | 0 – 2 | | 0 – 1 | | 0 – 4 | |

| Table S3. RQS Ratings and average rating per item | | | | | | | | | | | | | | | | | |
| --- | --- | --- | --- | --- | --- | --- | --- | --- | --- | --- | --- | --- | --- | --- | --- | --- | --- |
| **Author** | **Image Protocol** | **Multiple Segmentations** | **Phantom Study** | **Multiple Time Points** | **Feature Reduction** | **Non Radiomics** | **Biological Correlates** | **Cut-off** | **Discrimination and Resampling** | **Calibration** | **Prospective** | **Validation** | **Gold Standard** | **Clinical Utility** | **Cost-effectiveness** | **Open Science** | **Total** |
| **Feng[18]** | 1 | 1 | 0 | 0 | 3 | 0 | 0 | 0 | 2 | 0 | 0 | 2 | 2 | 2 | 0 | 1 | 14 |
| **Zhang.R[19]** | 1 | 1 | 0 | 0 | 3 | 1 | 1 | 0 | 2 | 0 | 0 | 2 | 2 | 2 | 0 | 1 | 16 |
| **Chong[20]** | 1 | 1 | 0 | 0 | 3 | 0 | 0 | 1 | 2 | 0 | 0 | 2 | 2 | 2 | 0 | 1 | 15 |
| **Zhu YJ[21]** | 1 | 1 | 0 | 0 | 3 | 1 | 1 | 1 | 2 | 0 | 0 | 2 | 2 | 0 | 0 | 1 | 15 |
| **Willson G[22]** | 1 | 1 | 0 | 0 | 3 | 1 | 0 | 1 | 2 | 0 | 0 | 2 | 2 | 0 | 0 | 1 | 14 |
| **Zhang Y[23]** | 1 | 1 | 0 | 0 | 3 | 1 | 0 | 1 | 2 | 0 | 0 | 2 | 2 | 2 | 0 | 1 | 16 |
| **Nebbia[24]** | 1 | 1 | 0 | 0 | 3 | 0 | 0 | 0 | 2 | 0 | 0 | 2 | 2 | 0 | 0 | 1 | 12 |
| **Chen Y[25]** | 1 | 1 | 0 | 0 | 3 | 0 | 0 | 1 | 2 | 0 | 0 | 2 | 2 | 0 | 0 | 1 | 13 |
| **Dai[26]** | 1 | 0 | 0 | 0 | 3 | 0 | 0 | 0 | 2 | 0 | 0 | 2 | 2 | 0 | 1 | 1 | 12 |
| **Meng[27]** | 1 | 1 | 0 | 0 | 3 | 0 | 0 | 0 | 2 | 0 | 0 | 2 | 2 | 2 | 0 | 1 | 14 |
| **Yang Y[28]** | 1 | 1 | 0 | 0 | 3 | 1 | 0 | 1 | 2 | 0 | 0 | 2 | 2 | 2 | 0 | 1 | 16 |
| **Qu C[29]** | 1 | 1 | 0 | 0 | 3 | 1 | 0 | 1 | 2 | 0 | 0 | 2 | 2 | 2 | 0 | 1 | 16 |
| **Jiang T[30]** | 1 | 1 | 0 | 0 | 3 | 1 | 0 | 1 | 2 | 0 | 0 | 2 | 2 | 2 | 0 | 1 | 16 |
| **Gao L[31]** | 1 | 1 | 0 | 0 | 3 | 1 | 0 | 1 | 2 | 0 | 0 | 2 | 2 | 2 | 0 | 1 | 16 |
| **Tian Y[32]** | 1 | 1 | 0 | 0 | 3 | 1 | 0 | 0 | 2 | 0 | 0 | 4 | 2 | 2 | 0 | 1 | 17 |
| **Defined range** | 0 – 2 | 0 – 1 | 0 – 1 | 0 – 1 | -3 – 3 | 0 – 1 | 0 – 1 | 0–1 | 0 – 2 | 0 – 1 | 0 – 7 | -5 – 5 | 0 – 2 | 0 – 2 | 0 – 1 | 0 – 4 | 222 |
